# The Integrated Effect of Microbial Inoculants and Biochar Types on Soil Biological Properties, and Plant Growth of Lettuce (*Lactuca sativa* L.)

**DOI:** 10.3390/plants11030423

**Published:** 2022-02-03

**Authors:** Hua Ma, Vyacheslav Shurigin, Dilfuza Jabborova, Jeane Aril dela Cruz, Thomas Edison dela Cruz, Stephan Wirth, Sonoko Dorothea Bellingrath-Kimura, Dilfuza Egamberdieva

**Affiliations:** 1 School of Life Sciences, Chongqing University, Chongqing 401331, China; 2 Faculty of Biology, National University of Uzbekistan, Tashkent 100174, Uzbekistan; slaventus87@inbox.ru; 3 Institute of Genetics and Plant Experimental Biology, Academy of Sciences of Uzbekistan, Tashkent 111226, Uzbekistan; dilfuzajabborova@yahoo.com; 4 Department of Biological Sciences, College of Science, University of Santo Tomas, Manila 1008, Philippines; jadelacruz@ust.edu.ph (J.A.d.C.); tedelacruz@ust.edu.ph (T.E.d.C.); 5 Fungal Biodiversity, Ecogenomics, and Systematics (FBeS) Group, Research Center for the Natural and Applied Sciences, University of Santo Tomas, Manila 1008, Philippines; 6 Leibniz Centre for Agricultural Landscape Research (ZALF), 15374 Müncheberg, Germany; swirth@zalf.de (S.W.); belks@zalf.de (S.D.B.-K.); 7 Faculty of Life Science, Humboldt University of Berlin, 14195 Berlin, Germany

**Keywords:** pyrolysis biochar, plant biomass, nutrient uptake, soil enzyme activities, nitrogen, phosphorus

## Abstract

Numerous reports confirm the positive effect of biochar application on soil properties and plant development. However, the interaction between root-associated beneficial microbes and different types of biochar is not well understood. The objective of this study was to evaluate the plant growth of lettuce after the application of three types of biochar in loamy, sandy soil individually and in combination with plant-beneficial microbes. Furthermore, total microbial activity in rhizosphere soil of lettuce was measured by means of fluorescein diacetate (FDA) hydrolase and enzyme activities linked to carbon, nitrogen, and phosphorus cycling. We used three types of biochar: (i) pyrolysis char from cherry wood (CWBC), (ii) pyrolysis char from wood (WBC), and (iii) pyrolysis char from maize (MBC) at 2% concentration. Our results showed that pyrolysis biochars positively affected plant interaction with microbial inoculants. Plant dry biomass grown on soil amended with MBC in combination with *Klebsiella* sp. BS13 and *Klebsiella* sp. BS13 + *Talaromyces purpureogenus* BS16aPP inoculants was significantly increased by 5.8% and 18%, respectively, compared to the control plants. Comprehensively, interaction analysis showed that the biochar effect on soil enzyme activities involved in N and P cycling depends on the type of microbial inoculant. Microbial strains exhibited plant growth-promoting traits, including the production of indole 3-acetic-acid and hydrogen cyanide and phosphate-solubilizing ability. The effect of microbial inoculant also depends on the biochar type. In summary, these findings provide new insights into the understanding of the interactions between biochar and microbial inoculants, which may affect lettuce growth and development.

## 1. Introduction

Biochar is produced from agricultural residues or other bio-waste, e.g., wood chips or sewage sludge, by pyrolysis under low or in the absence of oxygen [1], and is considered to improve soil health and crop productivity and discussed as a strategy for carbon sequestration [2,3]. Several reports are available on the positive effect of biochar application, produced from different feedstock on soil cation exchange capacity [4], soil enzyme activity [5,6], soil water holding capacity [7], and soil organic matter contents [8]. Moreover, biochar application enhanced plant growth of various crops such as pepper and tomato [9], soybean and chickpea [10,11], maize [12], and wheat [13]. Such positive effects by biochar application were often explained by enhanced diversity of soil microbial communities, which exhibits plant-beneficial traits and their improved activities involved in nutrient cycling [9,14,15,16,17]. In addition, soil microbes directly promote plant health, nutrient uptake, and plant tolerance to biotic and abiotic stresses through the synthesis of various enzymes, phytohormones, and other metabolites [10,15]. Due to carbon and nutrient concentrations in biochar types, soil microbial activity may vary in response to biochar addition [18]. Thus, the investigation of the impact of soil amendments with biochar on soil microbial community and their interactions with biochar types is of great importance. Soil enzymes secreted and synthesized by microbes play an essential role in the mineralization of soil organic matter, and they are sensitive to abiotic and biotic factors. In some reports, biochar improved soil enzyme activities involved in C and N cycles and overall microbial activity [19], while other studies observed an inhibition of soil fluorescein diacetate hydrolase, protease, and glycosidase activities [20]. 

Plant growth and nutrition have been reported to be improved by the combination of biochar and beneficial microbes. For example, the plant beneficial bacteria *Bacillus amyloliquefaciens* combined with biochar application produced from compost showed a positive effect on spinach [21]. Corresponding findings are available for *Paenibacillus polymyxa*, combined with wood biochar on switchgrass [22], and for *Pseudomonas fluorescens* and pinewood biochar on cucumber [23]. The plant-beneficial microbes *Talaromyces* and *Klebsiella* strains used in this study were isolated from the rhizosphere soil of lettuce. They showed several plant growth-promoting traits, including phosphate solubilization activity, and antagonized the plant pathogen *Fusarium oxysporum*, the causative agent of *Fusarium* wilt [24]. 

Lettuce (*Lactuca sativa* L.) of the Asteraceae family is native to the eastern Mediterranean region and western Asia, as well as South Europe. Lettuce provides a good source of minerals and biologically active compounds [25,26] and is cultivated worldwide. However, studies about the impacts of microbial inoculants combined with biochar amendments for improving the growth of lettuce are rare or missing.

There is evidence that plant growth, nutrient acquisition, soil biochemical processes, and microbial communities respond differently to biochar amendments depending on the feedstock used and the production technology [27]. It is evidence that soil microbial activity plays an important role in the mineralization of nutrients in the soil through extracellular enzymes [28]. 

However, the interaction between root-associated beneficial microbes and amendments with different biochar types is not well understood. Our study hypothesized that different types of biochar affect soil–plant–microbe interactions by improving soil biological properties in the plant root system. Here we investigated the effect of three different biochar types produced from maize, black cherry, or wood on the growth of lettuce in combination with an inoculation of plant growth-promoting bacteria and fungi. The objectives of this study were: (1) to evaluate the response of growth of lettuce to the application of three types of biochar applied in loamy, sandy soil individually and in combination with plant-beneficial microbes; (2) to determine the total microbial activity as measured by fluorescein diacetate (FDA) hydrolase in the rhizosphere soil of lettuce; and (3) to analyze rhizosphere enzyme activities linked to carbon, nitrogen, and phosphorus cycling.

## 2. Results

### 2.1. Plant Dry Biomass

**Figure 1:**
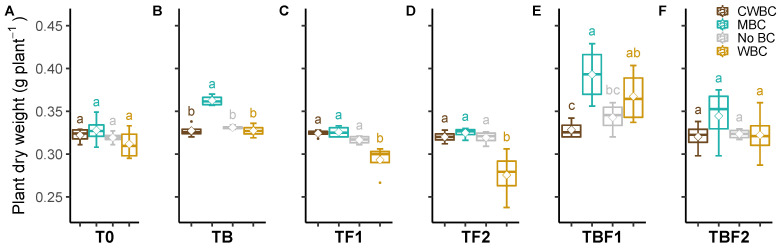
Effect of microbial inoculants on plant dry weight after biochar application. Un-inoculated control (T0, (**A**)), inoculated with *Klebsiella* sp. BS13 (TB, (**B**)), inoculated with *Talaromyces purpureogenus* BS16aPP (TF1, (**C**)), inoculated with *Talaromyces calidicanius* RS10bPP (TF2, (**D**)), inoculated with *Klebsiella* sp. BS13 + *Talaromyces calidicanius* RS10bPP (TBF1, (**E**)), and inoculated with *Klebsiella* sp. BS13 + *Talaromyces purpureogenus* BS16aPP (TBF2, (**F**)). Quantiles are shown at the top and bottom of the box. Max and min values are indicated by the bars. The lines within the box indicate the median values. The transparent dot indicates the observation value. Letters above the bars indicate the significance level at *p* < 0.05 by LSD. CWBC—black cherry wood biochar, MBC—pyrolysis biochar from maize, No BC—without biochar, WBC—pyrolysis biochar from wood.

The plant biomass of lettuce responded differently to the applied biochar type (CWBC, black cherry wood biochar; MBC, pyrolysis biochar from maize; WBC, pyrolysis biochar from wood). There was a slight increase in plant biomass grown in soil amended with MBC, but no effect was observed in soil with CWBC or WBC addition. The effect of microbial inoculants on the plant dry weight of lettuce showed that TB (inoculated with *Klebsiella* sp. BS13) and TBF1 (inoculated with *Klebsiella* sp. BS13 + *Talaromyces calidicanius* RS10bPP) slightly increased dry biomass compared to un-inoculated plants. The other treatments TF1 (inoculated with *Talaromyces purpureogenus* BS16aPP), TF2 (inoculated with *Talaromyces calidicanius* RS10bPP), and TBF2 (inoculated with *Klebsiella* sp. BS13 + *Talaromyces purpureogenus* BS16aPP), did not show any stimulation of plant growth (Figure 1A).

The effects of biochar types (CWBC, WBC, and MBC) combined with microbial inoculants on the dry weight of lettuce was investigated. No differences of dry plant biomass between plants inoculated with microbes (TB, TF1, TF2, TBF1, and TBF2) grown in soil amended with CWBC were observed (Figure 1B). In soil amended with WBC, plants inoculated with TBF1 showed a significant (*p* < 0.05) increase (18%) in plant biomass compared to un-inoculated plants. In contrast, TF1 and TF2 decreased plant growth under WBC compared to un-inoculated plants.

Compared to other biochar applications in soil, WBC had a beneficial effect on plant interactions with microbial inoculants. In soil amended with MBC, the dry plant biomass of lettuce inoculated with TB and TBF1 were significantly (*p* < 0.05) increased by 11 and 20% compared to un-inoculated plants (Figure 1C). There were no effects of TF1 and TF2, except TBF1 increased plant growth slightly. 

In general, our results indicate that MBC positively affects plant interaction with microbial inoculants. Moreover, dry plant biomass grown in soil amended with MBC combined with TB and TBF1 was increased compared to the control plants and CWBC and WBC. 

The interactions of biochar × microbes on the plant dry weight were significant (*p* < 0.01, Table 1). The plant dry weight in the MBC treatment was higher for each microbial treatment. The plant dry weight of WBC treatment was lower in T0, TF1, and TF2 treatments, but higher in TBF1 treatment. In addition, all biochar treatments showed a higher plant dry weight in TBF1 treatment. 

### 2.2. Plant Beneficial Traits of Microbial Inoculants

All three microbial inoculants were tested for their ability to produce HCN (hydrogen cyanide), IAA (3-Indoleacetic acid), and solubilize inorganic phosphorus. *Klebsiella* sp. BS13 and *Talaromyces purpureogenus* BS16aPP produced 3.4 and 2.6 µg/mL IAA, respectively, and showed phosphate solubilization activity. HCN production was observed only in *Klebsiella* sp. BS13. *Talaromyces calidicanius* RS10bPP showed low IAA production activity (1.9 µg/mL IAA) and was negative for phosphate solubilization and HCN activities. Based on the above data, it was found that the bacterial strain BS13 had the best growth-promoting traits. 

### 2.3. Soil Enzymes

**Figure 2:**
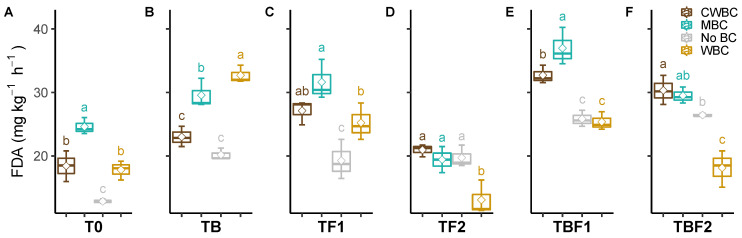
Effect of microbial inoculants on soil FDA hydrolytic activity after biochar application. Treatment abbreviations—see Figure 1. Quantiles are shown at the top and bottom of the box. Max and min values are indicated by the bars. The lines within the box indicate the median values. The transparent dot indicates the observation value. Letters above the bars indicate the significance level at *p* < 0.05 by LSD.

Generally, biochar (CWBC, WBC, and MBC) enhanced the soil FDA hydrolytic activity significantly without any microbe inoculation (Figure 2). While TB, TF1, TBF1, and TBF2 were inoculated, the soil FDA hydrolytic activity was improved under three types of biochar application, except WBC treatment with TBF2 inoculation. The interactions of biochar × microbes on the activities of soil FDA hydrolase protease, AKP, and ACP were significant (*p* < 0.001, Table 1). The effect of WBC treatment on the FDA hydrolytic activity was the highest in the TB treatment but the lowest in the TF2 treatment. The biochar effect on the FDA hydrolytic activity tended to be lower in TF2 treatment.

**Figure 3:**
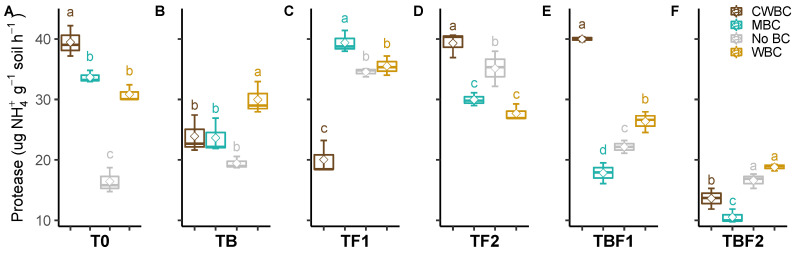
Effect of microbial inoculants on soil protease activity after biochar application. Treatment abbreviations—see Figure 1. Quantiles are shown at the top and bottom of the box. Max and min values are indicated by the bars. The lines within the box indicate the median values. The transparent dot indicates the observation value. Letters above the bars indicate the significance level at *p* < 0.05 by LSD.

The biochar (CWBC, WBC, and MBC) effect on the soil protease activity was significantly higher than the control without microbe inoculation (Figure 3). Interestingly, this positive effect was eliminated complately while various microbes were inoculated. For instance, CWBC and MBC showed no effect on the soil protease activity except WBC under TB inoculation. On the other hand, WBC showed no effect but CWBC and MBC showed a negative and positive effect, respectively on the soil protease activity under TF1 inoculation. The soil protease activity of three biochar applied treatments was obviously decreased under TBF2 inoculation in comparison to T0. In addition, the effect of CWBC treatment on the protease activity was the highest in T0, TF2, and TBF1 treatment but the lowest in TF1 treatment. Biochar effect on the protease activity was higher in T0 and TF1 treatment, but lower in TBF2 treatment.

**Figure 4:**
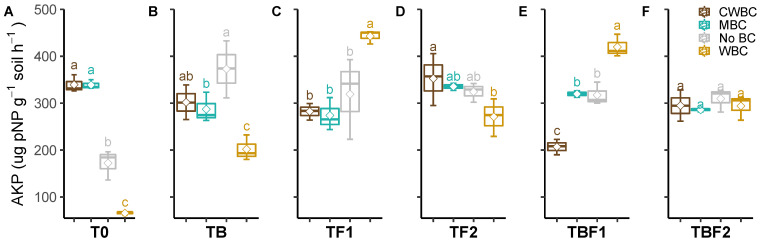
Effect of microbial inoculants on soil alkaline phosphomonoesterase activity after biochar application. Treatment abbreviations—see Figure 1. Quantiles are shown at the top and bottom of the box. Max and min values are indicated by the bars. The lines within the box indicate the median values. The transparent dot indicates the observation value. Letters above the bars indicate the significance level at *p* < 0.05 by LSD.

CWBC and MBC enhanced, but WBC decreased, the soil AKP activity without inoculation (Figure 4). MBC and WBC showed significantly lower AKP activity than the control under TB inoculation. The biochar treatments and control showed a similar tendency of the soil AKP activity in TF1 and TBF1, TF2, and TBF2. WBC indicated higher AKP activity than CWBC, MBC, and control under TF1 and TBF1 inoculation. There was no significant difference between biochar treatments and control under TF2 and TBF2 inoculation. The interaction of biochar × microbes on the AKP activity was complicated. Each biochar effect on the AKP activity showed an increase or decrease in microbe treatments.

**Figure 5:**
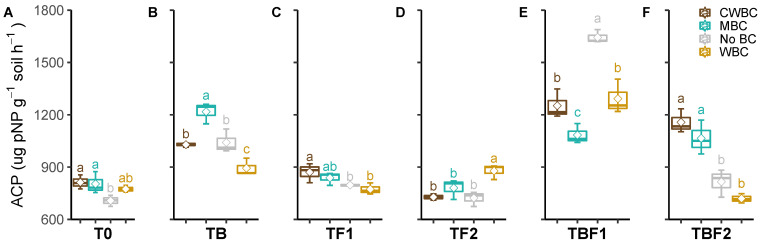
Effect of microbial inoculants on the soil acidic phosphomonoesterase activity after biochar application. Treatment abbreviations—see Figure 1. Quantiles are shown at the top and bottom of the box. Max and min values are indicated by the bars. The lines within the box indicate the median values. The transparent dot indicates the observation value. Letters above the bars indicate the significance level at *p* < 0.05 by LSD.

CWBC and MBC showed a significantly positive effect on the soil ACP activity without inoculation (Figure 5). MBC enhanced, but WBC decreased, the ACP activity under TB inoculation. CWBC and WBC indicated the highest ACP activity under TF1 and TF2 inoculation, respectively. No significant effect of the other two biochar treatments was observed on the ACP activity under TF1 and TF2 inoculation, in comparison to the control. Conversely, the control showed the highest ACP activity under TBF1 inoculation. CWBC and MBC indicated significantly higher ACP activity than WBC and the control under TBF2 inoculation. The interaction of biochar × microbes on the ACP activity was lower since the biochar effect on the ACP activity showed a similar tendency in microbe treatments. Comprehensively, interaction analysis showed the biochar effect on soil enzyme activities depends on the microbe type, and the microbe effect also depends on the biochar type. 

## 3. Discussion

The present study demonstrated positive interactions of biochar amendments with microbial inoculants, associated with beneficial effects on lettuce growth and soil biological activity in the rhizosphere. The biomass of lettuce, both un-inoculated and inoculated with microbes, were higher for soil amended with MBC as compared to plants grown in soil without biochar or amended with CWBC and WBC. Organic carbon and minerals in biochar provide additional nutrients to the soil that are readily available to plants, thus improving their nutritional status and development [29,30]. Several studies reported induced changes in nutrient availability after biochar application, providing additional sources of N, P, and carbon sources for microbes associated with plant roots [31]. The improvement of plant-associated microbial activity in soil amended with biochar was reported in several studies for various crops [32,33,34]. It is well documented that biochar carbon-rich material provides favorable conditions for the proliferation of root-associated microbes involved in carbon, nitrogen, and phosphorus cycles in soil and thus increase nutrient availability for plants [10]. Furthermore, biochar enhanced the diversity of beneficial microbes which produce various metabolites, such as phytohormones, hydrolytic enzymes, antifungal compounds, and siderophores, which promote plant growth and stress tolerance [35]. Biochar produced from cornhusk showed significant effects on bacterial diversity, whereas dominant genus *Bacillus*, plant-beneficial bacteria, were abundant [36]. In another study, soil amended with straw biochar increased the abundance of the phosphate-solubilizing bacterial community and their survival [37]. Hale et al. [38] observed a high survival rate of plant-beneficial bacteria that produced the phytohormone auxin after biochar application. 

Based on our results, we found that *Klebsiella* sp. BS13 and *T. purpureogenus* BS16aPP had the ability to produce IAA and solubilize phosphate. An early study also showed other beneficial properties, which included the ability to solubilize phosphate as indicated by their phosphate solubilization (PS) index [BS13, PSI = 2.43; BS16aPP, PSI = 2.66] and their ability to antagonize the plant pathogen *Fusarium oxysporum*, the agent that causes *Fusarium* wilt, an impairing disease in economic crops [24]. 

This fits the general view that beneficial root-associated bacteria stimulate plant growth through several traits, such as the production of phytohormones, siderophores, or phosphate [39,40]. Here, however, it is added that the combination of biochar with plant growth-promoting microbes specifically improve the growth and development of lettuce, and the biochar effect on the plant dry weight depends on microbe type. 

We have also observed changes in soil enzyme activities by biochar application and treatment with bacterial inoculants. Soil FDA hydrolytic activity indicates overall soil microbial activity. The highest soil microbial activity, as observed by FDA hydrolytic activity, was observed in soil amended with WBC and combined with *Klebsiella* sp. BS13 (TB), as compared to the other treatments and control soil without biochar application. An increased FDA activity was recorded in soil amended with biochar under soybean [41] and okra [42], which was explained as an enhanced organic matter in the soil for metabolic activity of microbes. Biochar enriched with nutrients provides benefits, supports microbial proliferation in the root system, and protects from various abiotic stresses [43,44]. Moreover, the biochar pores colonized by introduced microbial inoculants are protected from various abiotic factors [45]. Our results agree with previous findings by Ma et al. [4], who reported an increased FDA hydrolase activity in soil under soybean amended with biochar produced from black cherry wood. In another study [46], a higher soil FDA hydrolytic activity by microbial inoculation, compared to un-inoculated plants, was also reported. It is stated that soil organic matter input by biochar application is responsible for prospering soil biological activities, especially in the soil–plant system [47,48]. Other studies reported contrasting results, where soil FDA activity under carrot was inhibited by softwood biochar application [49]. Li et al. [50] also observed a decreased microbial biomass in soil amended with a higher dose of bamboo biochar (40 t/ha). It was explained by a reduced mineralization rate of soil organic carbon after the addition of a high amount of wood biochar [51]. These findings suggest that the response of soil microbial activity to biochar addition depends on environmental factors, biochar type, and rate of application.

Phosphatases play a vital role in P cycles. Alkaline phosphomonoesterase activity was promoted in biochar treated soil, and its activity was increased by microbial inoculants TF1 and TBF1 combined with CWBC. However, no changes were found in the other treatments. There were also changes in soil acidic phosphomonoesterase activity, as it was increased by all types of biochar, combined with microbial inoculants TB and TBF1. It is known that plant-associated microbes are involved in P mineralization, increasing the availability of P for plant uptake [52,53]. Moreover, *Klebsiella* sp. BS13 (TB) produced HCN, which is involved in the indirect increase of phosphate availability [54]. Furthermore, soil protease activity increased after the application of all types of biochar, CWBC, MBC, and WBC, indicating an improved physiological status of the microbial communities related to C, N, and P cycling activities [27,55]. Accordingly, Wang et al. [56] observed increased enzyme activities involved in C and N cycles in soil amended with maize biochar.

## 4. Materials and Methods

### 4.1. Plant, Soil and Biochars

Soil samples were taken at the field station of the Leibniz Centre for Agricultural Landscape Research, Müncheberg, Germany, in 2019. The soil is a loamy sand (Luvisol) with 7% clay, 19% silt, and 74% sand, C org—0.6%, total N—0.07%, P—0.03%, K—1.25%, and Mg—0.18%, the pH was 6.2 [11]. Three biochar types were used in this study: (i) black cherry wood biochar (CWBC), (450 °C for 30 min); (ii) pyrolysis biochar from maize (MBC), (600 °C for 30 min); (iii) pyrolysis biochar from wood (WBC), (850 °C for 30 min), (Table 2, [4,57]. These three biochars were chosen due to their potential applications in the field or greenhouse for crop production. Cherry wood biochar is a biochar product obtainable in Germany. Maize cob is a very common material for farmers to obtain. Wood biochar is derived from mixed woodnot separated by type when producing biochar. The different types of biochar were acquired from the Leibniz-Institute for Agrartechnik Potsdam-Bornim e.V. (ATB), Germany.

### 4.2. Microorganisms

The plant growth-stimulating bacteria and fungi were previously isolated from soil collected at a lettuce farm (16.4580° N, 120.5878° E) in La Trinidad, Benguet Province, Northern Philippines. These were identified as *Klebsiella* sp. (BS13), *Talaromyces calidicanius* (RS10bPP), and *Talaromyces purpureogenus* (BS16aPP) and showed the ability to solubilize phosphate and to antagonize the pathogenic fungus *Fusarium oxysporum* [24]. 

### 4.3. Plant Growth Experiment

The biochar was used at 2% concentration as a soil amendment. Pots (d = 0.16 m, v = 2016 cm^3^) were filled with 1 kg of soil and mixed with crushed chars. Sterilized lettuce seeds (10% *v*/*v* NaOCl and 70% ethanol) were germinated in a dark room at 25 °C for three days. The strain *Klebsiella* sp. BS13 was grown in Tryptic Soy Broth (TSB) (Difco Laboratories, Detroit, MI, USA) for 48 h at 28 °C. To approach a final density of bacteria at 10^8^ CFU mL^−1^, the culture suspension was re-suspended in PBS. The fungal isolates *Talaromyces calidicanius* RS10bPP and *Talaromyces purpureogenus* BS16aPP were grown in PDA agar plates (Difco Laboratories, Detroit, MI, USA) at 28 °C for five days. The spores of the fungal isolates were washed on a PDA plate with sterile water containing two drops of Tween 80. The spores were counted with a hemocytometer, and the suspension was diluted to a concentration of 10^7^ spores mL^−1^. Germinated seeds were immersed into bacterial and/or fungal suspensions and transferred to pots.

The following treatments were set up:

1. T0: un-inoculated control plants grown in soil; (a) without biochar, (b) with CWBC, (c) with WBC, (d) with MBC;
2. TB: inoculated plants with *Klebsiella* sp. BS13 and grown in soil; (a) without biochar, (b) with CWBC, (c) with WBC, (d) with MBC;
3. TF1: inoculated plants with *Talaromyces purpureogenus* BS16aPP and grown in soil; (a) without biochar, (b) with CWBC, (c) with WBC, (d) with MBC;
4. TF2: inoculated plants with *Talaromyces calidicanius* RS10bPP and grown in soil; (a) without biochar, (b) with CWBC, (c) with WBC, (d) with MBC;
5. TBF1: inoculated plants with *Klebsiella* sp. BS13 + *Talaromyces calidicanius* RS10bPP and grown in soil; (a) without biochar, (b) with CWBC, (c) with WBC, (d) with MBC;
6. TBF2: inoculated plants with *Klebsiella* sp. BS13 + *Talaromyces purpureogenus* BS16aPP and grown in soil; (a) without biochar, (b) with CWBC, (c) with WBC, (d) with MBC.

This study used a randomized complete block design, comprising four replications in four blocks, each including all six treatments. The treatments were distributed randomly in each block.

Each pot was sown with three seeds; one-week seedlings were thinned to one plant per pot. Plants were grown for 30 days under greenhouse conditions at a temperature of 24 °C/16 °C (day/night) and a humidity of 50–60%. At harvest, the roots and shoots were separated, washed, and oven-dried at 70 °C for 48 h, and dry weight was determined. 

### 4.4. The Plant Beneficial Traits and Colonization Ability of Microbial Inoculants 

The HCN produced by bacterial isolates was tested on a Tryptic Soy Agar (TSA) medium for bacteria and a Potato Dextrose Agar (PDA) medium for fungi. The color change of filter paper saturated with 1% picric acid and 2% sodium carbonate solutions was measured [58]. The IAA produced (indole 3-acetic acid) by microbial strains was studied using the method of Bano and Musarrat [59], evaluating IAA production by detecting pink color after 30 min. The qualitative analysis of the phosphate solubilization potential of microbial inoculants was measured in vitro by determining available soluble phosphate in Pikovskaya’s medium [60], supplemented with tri-calcium phosphate. The colonies of tested bacteria and fungi were inoculated at the center of the agar plate and incubated at 28 °C. After four days, the diameters of the appearing ring by dissolved phosphate around the colonies were measured. Other beneficial properties of the microbial inoculants were reported earlier [24]. 

### 4.5. Soil Enzyme Activities

The FDA hydrolytic activity was determined by the method of Green et al. [61]. In total, 0, 0.001, 0.005, 0.05, and 0.15 mg of fluorescein was used for standard curve preparation. The method of Tabatabai and Bremner [62] was used to determine acid (ACP) and alkaline phosphomonoesterase (AKP) activities in soil. The produced p-nitrophenol (p-NP) in the assays was computed by a p-NP calibration curve (400 nm wavelength) using a Lambda 2 UV-VIS spectrophotometer (Perkin Elmer) [63]. Protease activity was measured by the method of Ladd and Butler [64]. 

The soil around the roots was collected; the particle size of the soil was confirmed to be less than 2 mm. Then, the soil was air-dried for further nutrient analysis. The dry combustion method and an elemental determinator (TruSpec CNS) (Nelson and Sommers 1982) were used to determine soil carbon (Ct) and nitrogen (Nt) contents. Soil P and K contents were analyzed with an ICP-OES (iCAP 6300 Duo) via the Mehlich-3 extraction method. 

### 4.6. Statistical Analysis

The data were processed using the package “dplyr” of the open-source statistical language R v1.4.1717 (R Studio, Boston, MA, USA). The one-way analysis of variance (ANOVA) and the multiple comparisons of the means were performed by the package “agricolae” for a least significant difference (LSD, *p* = 0.05) test. The figures were plotted using the package “ggplot2”, and the plot panels were aligned using the package “ggpubr”. The package “HH” was used for analyzing the interactions between biochar and microbes.

## 5. Conclusions

Our findings demonstrate the positive synergistic effects of biochar amendments and the inoculation of plant-beneficial microbes on plant growth of lettuce and on soil enzyme activities in the rhizosphere. In general, biochar addition in soil combined with bacterial and fungal inoculants promoted the highest lettuce biomass. Indications were thus provided that the biochar effect on plant dry weight depends on the type of microbial inoculant. Microbial strains showed plant growth-improving traits, including the production of phytohormone IAA and hydrogen cyanide, and they also showed phosphate-solubilizing ability. Comprehensively, an interaction analysis showed that the biochar effect on soil enzyme activities involved in N and P cycling depends on the type of microbial inoculants. Furthermore, the microbe effect also depends on the type of biochar amendment. Taken together, these findings provide new insights into understanding the interactions between biochar and microbial inoculant, which may affect lettuce growth and development.

## Figures and Tables

**Table 1:** Interaction effects of biochar and microbial inoculants on the plant dry weight and the activities of soil fluorescein diacetate (FDA) hydrolase, protease, alkaline phosphomonoesterase (AKP), and acidic phosphomonoesterase (ACP).

Interaction Effects	Plant Dry Weight	Soil FDA	Soil Protease	Soil AKP	Soil ACP
Biochar	***	***	***	ns	***
Microbes	***	***	***	***	***
Biochar × Microbes	**	***	***	***	***

Interaction effects of biochar and microbes on plant dry weight and soil enzymes. Significance denoted by ** *p* < 0.01, *** *p* < 0.001, ns: no significance.

**Table 2:** Characterization of chars.

Material	C %	N %	P (g/kg)	K (g/kg)	pH
CWBC-char	41.5	0.37	3.26	11,5	8.41
MBC-char	75.16	1.65	5.26	31.12	9.89
WBC-char	77.62	0.72	1.24	7.8	9.35

CWBC—black cherry wood biochar; MBC—maize biochar; WBC—wood biochar [4,57].

## References

[1] Lehmann J., Rillig M.C., Thies J., Masiello C.A., Hockaday W.C., Crowley D. (2011). Biochar effects on soil biota—A review. Soil Biol. Biochem..

[2] Ippolito J.A., Laird D.A., Busscher W.J. (2012). Environmental benefits of biochar. J. Environ. Qual..

[3] Biederman L.A., Harpole W.S. (2013). Biochar and its effects on plant productivity and nutrient cycling: A meta-analysis. GCB Bioenergy.

[4] Novak J.M., Busscher W.J., Laird D.L., Ahmedna M., Watts D.W., Niandou M.A. (2009). Impact of biochar amendment on fertility of a southeastern coastal plain soil. Soil Sci..

[5] Ma H., Egamberdieva D., Wirth S., Li Q., Omari R.A., Hou M., Bellingrath-Kimura S.D. (2019). Effect of biochar and irrigation on the interrelationships among soybean growth, root nodulation, plant P uptake, and soil nutrients in a sandy field. Sustainability.

[6] Ma H., Egamberdieva D., Wirth S., Bellingrath-Kimura S.D. (2019). Effect of biochar and irrigation on soybean-rhizobium symbiotic performance and soil enzymatic activity in field rhizosphere. Agronomy.

[7] Yu O.Y., Raichle B., Sink S. (2013). Impact of biochar on the water holding capacity of loamy sand soil. Int. J. Energy Environ. Eng..

[8] Chan K.Y., Van Zwieten L., Meszaros I., Downie A., Joseph S. (2007). Agronomic values of greenwaste biochar as a soil amendment. Aust. J. Soil Res..

[9] Graber E.R., Meller-Harel Y., Kolton M., Cytryn E., Silber A., David D.R., Tsechansky L., Borenshtein M., Elad Y. (2010). Biochar impact on development and productivity of pepper and tomato grown in fertigated soilless media. Plant Soil..

[10] Egamberdieva D., Wirth S., Behrendt U., Abd_Allah E.F., Berg G. (2016). Biochar treatment resulted in a combined effect on soybean growth promotion and a shift in plant growth promoting rhizobacteria. Front. Microbiol..

[11] Egamberdieva D., Li L., Ma H., Wirth S., Bellingrath-Kimura S.D. (2019). Soil amendments with different maize biochars, to varying degrees, improve chickpea growth under drought by improving symbiotic performance with *Mesorhizobium ciceri* and soil biochemical properties. Front. Microbiol..

[12] Islami T., Curitno B., Basuki N., Suryanto A. (2011). Maize yield and associated soil quality changes in cassava + maize intercropping system after 3 years of biochar application. J. Agric. Food Technol..

[13] Alburquerque J.A., Salazar P., Barrón V., Torrent J., del Campillo M.D., Gallardo A., Villar R. (2013). Enhanced wheat yield by biochar addition under different mineral fertilization levels. Agron. Sustain. Dev..

[14] Pietikäinen J., Kiikkila O., Fritze H. (2000). Charcoal as a habitat for microbes and its effects on the microbial community of the underlying humus. Oikos.

[15] Kolton M., Meller Harel Y., Pasternak Z., Graber E.R., Elad Y., Cytryn E. (2011). Impact of biochar application to soil on the root-associated bacterial community structure of fully developed greenhouse pepper plants. Appl. Environ. Microbiol..

[16] Egamberdieva D., Reckling M., Wirth S. (2017). Biochar-based inoculum of *Bradyrhizobium* sp. improves plant growth and yield of lupin (*Lupinus albus* L.) under drought stress. Eur. J. Soil Biol..

[17] Egamberdieva D., Wirth S., Shurigin V., Hashem A., Abd Allah E.F. (2017). Endophytic bacteria improve plant growth, symbiotic performance of chickpea (*Cicer arietinum* L.) and induce suppression of root rot caused by *Fusarium solani* under salt stress. Front. Microbiol..

[18] Song C., Zhu F., Carrión V.J., Cordovez V. (2020). Beyond Plant Microbiome Composition: Exploiting Microbial Functions and Plant Traits via Integrated Approaches. Front. Bioeng. Biotechnol..

[19] Lopes E.M.G., Reis M.M., Frazão L.A., da Mata Terra L.E., Lopes E.F., dos Santos M.M., Fernandes L.A. (2021). Biochar increases enzyme activity and total microbial quality of soil grown with sugarcane. Environ. Technol. Innov..

[20] Chintala R., Schumacher T.E., Kumar S.K., Malo D.D., Rice J.A., Bleakley B., Chilom G., Clay D.E., Julson J.L., Papiernik S.K. (2014). Molecular characterization of biochars and their influence on microbiological properties of soil. J. Hazard. Mater..

[21] Zafar-ul-Hye M., Tahzeeb-ul-Hassan M., Abid M., Fahad S., Brtnicky M., Dokulilova T., Datta R., Danish S. (2020). Potential role of compost mixed biochar with rhizobacteria in mitigating lead toxicity in spinach. Sci. Rep..

[22] Shanta N., Schwinghamer T., Backer R., Allaire S.E., Teshler I., Vanasse A., Whalen J., Baril B., Lange S., MacKay J. (2016). Biochar and plant growth promoting rhizobacteria effects on switchgrass (*Panicum virgatum* cv. Cave-in-Rock) for biomass production in southern Québec depend on soil type and location. Biomass Bioenergy.

[23] Nadeem S.M., Imran M., Naveed M., Khan M.Y., Ahmad M., Zahir Z.A., Crowley D.E. (2017). Synergistic use of biochar, compost and plant growth-promoting rhizobacteria for enhancing cucumber growth under water deficit conditions. J. Sci. Food Agric..

[24] Dela Cruz T.E.E., Din H.J.F., Aril-dela Cruz J.V. (2021). Microbes for Sustainable Agriculture: Isolation and Identification of Beneficial Soil- and Plant-Associated Microorganisms.

[25] Foteinis S., Chatzisymeon E. (2016). Life cycle assessment of organic versus conventional agriculture. A case study of lettuce cultivation in Greece. J. Clean. Prod..

[26] Anilakumar K.R., Harsha S.N., Mallesha S., Sharma R.K. (2017). Lettuce: A promising leafy vegetable with functional properties. Def. Life Sci. J..

[27] Gul S., Whalen J.K., Thomas B.W., Sachdeva V., Deng H.Y. (2015). Physico-chemical properties and microbial responses in biochar-amended soils: Mechanisms and future directions. Agric. Ecosyst. Environ..

[28] Wu H., Zeng G., Liang J., Chen J., Xu J., Dai J., Li X., Chen M., Xu P., Zhou Y. (2016). Responses of bacterial community and functional marker genes of nitrogen cycling to biochar, compost and combined amendments in soil. Appl. Microbiol. Biotechnol..

[29] Qayyum M.F., Steffens D., Reisenauer H.P., Schubert S. (2012). Kinetics of carbon mineralisation of biochars compared with wheat straw in three soils. J. Environ. Qual..

[30] Amini S., Ghadiri H., Chen C., Marschner P. (2016). Salt-affected soils, reclamation, carbon dynamics, and biochar: A review. J. Soils Sediments.

[31] Prendergast-Miller M.T., Duvall M., Sohi S.P. (2011). Localisation of nitrate in the rhizosphere of biochar-amended soils. Soil Biol. Biochem..

[32] Wang C., Alidousta D., Yng X., Isoda A. (2018). Effects of bamboo biochar on soybean root nodulation in multi-elements contaminated soils. Ecotoxicol. Environ. Saf..

[33] Egamberdieva D., Zoghi Z., Nazarov K., Wirth S., Bellingrath-Kimura S.D. (2020). Plant growth response of broad bean (*Vicia faba,* L.) to biochar amendment of loamy sand soil under irrigated and drought conditions. Environ. Sustain..

[34] Egamberdieva D., Shurigin V., Alaylar B., Ma H., Müller M.E.H., Wirth S., Reckling M., Bellingrath-Kimura S.D. (2020). The effect of biochars and endophytic bacteria on growth and root rot disease incidence of *Fusarium* infested narrow-leafed lupin (*Lupinus angustifolius* L.). Microorganisms.

[35] Quilliam R.S., Glanville H.C., Wade S.C., Jones D.L. (2013). Life in the ‘charosphere’-does biochar in agricultural soil provide a significant habitat for microorganisms?. Soil Biol. Biochem..

[36] Ogundeji A.O., Li Y., Liu X., Meng L., Sang P., Mu Y., Wu H., Ma Z., Hou J., Li S. (2021). Eggplant by grafting enhanced with biochar recruits specific microbes for disease suppression of *Verticillium* wilt. Appl. Soil Ecol..

[37] Zheng B.X., Ding K., Yang X.R., Wadaan M.A.M., Hozzein W.N., Peñuelas J., Zhu Y.G. (2019). Straw biochar increases the abundance of inorganic phosphate solubilizing bacterial community for better rape (*Brassica napus*) growth and phosphate uptake. Sci. Total Environ..

[38] Hale L., Luth M., Kenney R., Crowley D. (2014). Evaluation of Pinewood Biochar as a Carrier of Bacterial Strain *Enterobacter cloacae* UW5 for Soil Inoculation. Appl. Soil Ecol..

[39] Egamberdiyeva D. (2005). Plant growth promoting rhizobacteria isolated from calcisol soil in a semiarid region of Uzbekistan: Biochemical characterisation and effectiveness. Plant Nutr. Soil Sci..

[40] Cho S.T., Chang H.H., Egamberdieva D., Kamilova F., Lugtenberg B., Kuo C.H. (2015). Genome analysis of Pseudomonas fluorescens PCL1751: A rhizobacterium that controls root diseases and alleviates salt stress for its plant host. PLoS ONE.

[41] Van Zwieten L., Kimber S., Morris S. (2010). Effects of biochar from slow pyrolysis of papermill waste on agronomic performance and soil fertility. Plant Soil.

[42] Sarma B., Borkotoki B., Narzari R., Kataki R., Gogoi N. (2017). Organic amendments: Effect on carbon mineralization and crop productivity in acidic soil. J. Clean. Prod..

[43] Egamberdieva D., Alaylar B., Kistaubayeva A., Wirth S., Bellingrath-Kimura S.D. (2021). Biochar for improving soil biological properties and mitigating salt stress in plants on salt-affected soils. Comm. Plant Soil Sci..

[44] Iijima M., Yamane K., Izumi Y., Daimon H., Motonaga T. (2015). Continuous application of biochar inoculated with root nodule bacteria to subsoil enhances yield of soybean by the nodulation control using crack fertilization technique. Plant Prod. Sci..

[45] Głodowska M., Schwinghamer T., Husk B., Smith D. (2017). Biochar based inoculants improve soybean growth and nodulation. J. Agric. Sci..

[46] Fall D., Bakhoum N., Nourou Sall Zoubeirou A.M., Sylla S.N., Diouf D. (2016). Rhizobial inoculation increases soil microbial functioning and gum arabic production of 13-year-old *Senegalia senegal* (L.) Britton, trees in the north part of Senegal. Front. Plant Sci..

[47] Haque M.M., Rahman M.M., Morshed M.M., Islam M.S., Afrad M.S.I. (2019). Biochar on soil fertility and crop productivity. Agriculturists.

[48] Zhu X., Chen B., Zhu L., Xing B. (2017). Effects and mechanisms of biochar-microbe interactions in soil improvement and pollution remediation: A review. Environ. Pollut..

[49] Shoaf N.L. (2014). Biochar and vermicompost amendments in vegetable cropping systems: Impacts on soil quality, soil-borne pathogens and crop productivity. Master’s Thesis.

[50] Li Q., Lei Z., Song X., Zhang Z., Ying Y., Peng C. (2018). Biochar amendment decreases soil microbial biomass and increases bacterial diversity in Moso bamboo (*Phyllostachys edulis*) plantations under simulated nitrogen deposition. Environ. Res. Lett..

[51] Dempster D.N., Glesson D.B., Solaiman Z.M., Jones D.L., Murphy D.V. (2012). Decreased soil microbial biomass and nitrogen mineralisation with Eucalyptus biochar addition to a coarse textured soil. Plant Soil.

[52] Blackwell P., Krull E., Butler G., Herbert A., Solaiman Z. (2010). Effect of banded biochar on dryland wheat production and fertiliser use in south-western Australia: An agronomic and economic perspective. Aust. J. Soil Res..

[53] Masto R.E., Kumar S., Rout T.K., Sarkar P., George J., Ram L.C. (2013). Biochar from water hyacinth (*Eichhornia crassipes*) and its impact on soil biological activity. Catena.

[54] Rijavec T., Lapanje A. (2016). Hydrogen Cyanide in the Rhizosphere: Not Suppressing Plant Pathogens, but Rather Regulating Availability of Phosphate. Front. Microbiol..

[55] Shi S., Tian L., Nasir F., Bahadur A., Batool A., Luo S., Yang F., Wang Z., Tian C. (2019). Response of microbial communities and enzyme activities to amendments in saline-alkaline soils. Appl. Soil Ecol..

[56] Wang X., Song D.L., Liang G.Q., Zhang Q., Ai C., Zhou W. (2015). Maise biochar addition rate influences soil enzyme activity and microbial community composition in a fluvo-aquic soil. Appl. Soil Ecol..

[57] Reibe K., Götz K.P., Ross C.L., Doering T.F., Ellmer F., Ruess L. (2015). Impact of quality and quantity of biochar and hydrochar on soil collembola and growth of spring wheat. Soil Biol. Biochem..

[58] Castric P.A. (1975). Hydrogen cyanide, a secondary metabolite of Pseudomonas aeruginosa. Can. J. Microbiol..

[59] Bano N., Musarrat J. (2003). Characterization of a new *Pseudomonas aeruginosa* strain NJ-15 as a potential biocontrol agent. Curr. Microbiol..

[60] Pikovskaya R.I. (1948). Mobilization of phosphorous in soil in the connection with vital activity of some microbial species. Mikorobiologiya.

[61] Green V.S., Stott D.E., Diack M. (2006). Assay for fluorescein diacetate hydrolytic activity: Optimalization for soil samples. Soil Biol. Biochem..

[62] Tabatabai M.A., Bremner J.M. (1969). Use of p-nitrophenol phosphate for the assay of soil phosphatase activity. Soil Biol. Biochem..

[63] Acosta-Martínez V., Tabatabai M.A., Dick R.P. (2011). Phosphorus cycle enzymes. Methods of Soil Enzymology.

[64] Ladd J.N., Butler J.H.A. (1972). Short-term assays of soil proteolytic enzyme activities using proteins and dipeptide derivatives as substrates. Soil Biol. Biochem..

